# The hunter and the hunted—A 3D analysis of predator-prey interactions between three-spined sticklebacks (*Gasterosteus aculeatus*) and larvae of different prey fishes

**DOI:** 10.1371/journal.pone.0256427

**Published:** 2021-08-26

**Authors:** Jorrit Lucas, Albert Ros, Sarah Gugele, Julian Dunst, Juergen Geist, Alexander Brinker

**Affiliations:** 1 Fisheries Research Station Baden-Wuerttemberg, LAZBW, Langenargen, Germany; 2 Aquatic Systems Biology Unit, Department of Ecology and Ecosystem Management, Center of Life and Food Sciences Weihenstephan, Technical University of Munich, Freising, Germany; 3 University of Konstanz, Konstanz, Germany; University of Maine at Farmington, UNITED STATES

## Abstract

Predator-prey interactions play a key life history role, as animals cope with changing predation risk and opportunities to hunt prey. It has recently been shown that the hunting success of sticklebacks (*Gasterosteus aculeatus*) targeting fish larvae is dependent on both the size of the prey and the prior exposure of its species to stickleback predation. The purpose of the current study was to identify the behavioural predator-prey interactions explaining the success or failure of sticklebacks hunting larvae of three potential prey species [roach (*Rutilus rutilus*), perch (*Perca fluviatilis*) and whitefish (*Coregonus wartmannii*)] in a 3D environment. Trials were carried out for each prey species at four different size classes in a standardised laboratory setup and were recorded using a slow motion, stereo camera setup. 75 predator-prey interactions including both failed and successful hunts were subject to the analysis. 3D track analysis indicated that sticklebacks applied different strategies. Prey with less complex predator escape responses, *i*.*e*. whitefish larvae, were hunted using a direct but stealthy approach ending in a lunge, while the behaviourally more complex roach and perch larvae were hunted with a faster approach. A multivariate logistic regression identified that slow average speed and acceleration of the prey in the initial stages of the hunt increased the probability of stickleback success. Furthermore, predators adjusted their swimming direction more often when hunting larger whitefish compared to smaller whitefish. The results suggest that appropriate and adequately timed avoidance behaviours, which vary between prey species and ontogenetic stages, significantly increase the chances of outmanoeuvring and escaping stickleback predation. Small whitefish larvae can reach similar levels of swimming performance compared to older conspecifics, but display ineffective anti-predator behaviours, resulting in higher hunting success for sticklebacks. Thus, the development of appropriate anti-predator behaviours depending on size appears to be the crucial factor to escaping predation.

## Introduction

Predator-prey interactions play a dominant role in the life histories of wild animals, which experience changing predation risks and/or changing opportunities to hunt throughout their ontogeny [1]. In order to survive and to increase fitness, predatory animals have to: I) locate and identify both potential prey and predators [2]; II) select, capture, disarm, and consume their prey [2]; and III) perform adequate predator avoidance and/or defence when hunted [3, 4], all of which requires complex central processing of visual, chemical or auditory cues and tactile stimuli [5–8]. The detection of potential predators and prey also involves a range of passive and active strategies. For example, hunters may increase the possibility of an encounter with their prey through ambush or overt hunting, and prey species might increase their chances of survival by hiding or joining schools of multiple, alternately vigilant individuals [2]. Furthermore, predators and prey do not develop in isolation: their biotic interactions may actually be a main driver of evolution [9].

Selection for strategies used by predators and prey is driven by trade-offs of time and energy requirements against those of other important life-history components, like territorial defence of resources, mating and reproduction, and/or defence of vulnerable offspring [10]. There are several central processes which can optimize these strategies: I) animals may have predispositions in anxiety and boldness that increase vigilance or responsiveness; II) animals may have sensory or central “templates” that aid precociously predator detection; III) animals acquire knowledge of their prey and predators through experience. The first two mechanisms play an especially important and often decisive role in initial interactions between predator and prey. The latter mechanism will refine these responses with age in both protagonists to increase their chances of survival to reproductive ages [11, 12].

Predispositions that exist before contact with a relevant stimulus may optimize responses to certain types of predator in a stable environment [13]. However, they are less adaptive in a complex environment and this may be critical if a novel predator, e.g., an invasive species, emerges [14]. The native prey species might not recognize the new predators as a threat, resulting in an absence of appropriate evasive behaviour and increased mortality, potentially over several generations [14]. Furthermore, an individual’s interactions with the social and physical environment will change throughout its life history, not least as a direct function of size. Such ontogenetic changes promote selection for flexible mechanisms such as learning, in order to optimize decision-making and responses to the behaviour of possible predators or prey. The impact of predator-prey interactions has been well studied in aquatic ecosystems, especially in fishes [7, 15–18]. Predation affects all major aspects of fish life-history, including growth, age at reproduction and behaviour [19–22] and the study of hunting behaviour is highly pertinent to understanding the ecology of fish species [23].

As a common and successful species in aquatic ecosystems of the northern hemisphere and quick to adapt to laboratory conditions, the three-spined stickleback (*Gasterosteus aculeatus*) has become a model species in behavioural studies in recent decades [24, 25]. Their highly developed social behaviours and opportunistic lifestyle are well described [26–29]. The species is typically a predator of aquatic benthic invertebrates and zooplankton such as daphnia and chironomid species, and is preyed upon in turn by predatory fish like the northern pike (*Esox lucius*) and European perch (*Perca fluviatilis*) [28, 30]. As an opportunistic species, the ability of sticklebacks to adapt to different and multiple food sources plays a significant role across the different life stages [31]. It was recently shown that the success of stickleback predation on fish larvae depends upon the size and species of prey [32, 33]. Quantitative examination of predation on roach (*Rutilus rutilus*), perch (*Perca fluviatilis*) and whitefish (*Coregonus wartmannii*) larvae shows a particularly high toll on small-sized whitefish larvae which historically grew up in habitat without predators, in comparison to similar-sized larvae of the other two species which have co-evolved with predators in their environment [32, 34]. Sticklebacks have recently increased strongly in abundance in the pelagial of Lake Constance prompting concern that predation on whitefish larvae may be of ecological importance [34]. Stickleback hunting success has been shown to decrease with prey size and with increasing variation in predator avoidance strategies shown by prey [33], but it has not been analysed thus far whether these differences might be related to larval swimming performance as well.

Important performance criteria in predator-prey interactions include the response time of the prey, the speed and efficacy of evasive behaviour *vs*. hunting behaviour, and factors that prompt the predator to abort the hunt [33, 35, 36]. Questions about the decisions that predators and prey may or must make during a hunt can be addressed using detailed analysis of high-definition video recordings [37, 38]. To avoid the time-consuming complexity of tracking subjects in a natural 3-dimensional environment, previous experimenters have often forced animals to respond in a two-dimensional way by reducing the dimensional axis of depth to a minimum [39]. Such a setup is far from a natural setting in which both horizontal and vertical movements may be evident [40]. Recent developments in consumer grade high-frame-rate cameras and efficient tracking software have made analyses of more complex 3D tracks increasingly accessible for researchers [41–43].

In order to further understand the vulnerability of different fish larvae species and different sizes to predatory behaviours, the aim of this study was to describe differences in response times and swimming speed using 3D-tracking of both predator and prey. Hunts were evaluated stage by stage using variables like swimming speed, acceleration, turning angles and relative orientation of predator and prey, approach of the prey, prey capture and overall success or failure of the hunt. It was hypothesized that: I) swimming speed, acceleration and turning angles of prey strongly determine its susceptibility to predation by stickleback; and II) differences in these variables among the three tested fish species will explain different predation risks in a realistic exposure scenario. The results should increase the understanding of hunting behaviour and decision making in sticklebacks. In particular, this study will help identify key factors that influence hunting success of sticklebacks on fish larvae, with a focus on whitefish in their changing habitat at Lake Constance.

## Material and methods

### Experimental animals and keeping conditions

Adult three-spined sticklebacks were obtained from the Lake Constance, located north of the Alps (47°30’ N, 9°30’ E), using gill nets in May and June 2017. They were maintained in groups of 30–40 individuals in 65 L tanks with dimensions of 31 x 57 x 36 cm connected to a flow-through system (Kunststoff Spranger GmbH, Germany) supplied with clear aerated water that was pumped from Lake Constance at a depth of 30 m. Sticklebacks used in the experiments were free of *Schistocephalus solidus* parasites. Water temperature for sticklebacks ranged from 15–18°C. Roach larvae were bred in captivity and kept at a density of 200 fish per tank. Whitefish larvae (wild offspring) were hatched at a nearby hatchery (Fischbrutanstalt Langenargen) and perch larvae (wild offspring) were kindly provided by the Institute of Limnology, University of Konstanz (Germany). Individuals of all three prey species were reared from eggs and, thus, had never experienced hunting behaviour before the start of the experiment. The light-dark cycle was 12:12 with lights on at 7:00 h. The temperature of the rearing basins was maintained at 15–18°C for whitefish, 19°C for roach, and 20°C for perch (using 75 W thermostats, Eheim). For further details on hatching and feeding conditions in this experiment see Ros *et al*. [33].

### Experimental setup

All sticklebacks were acclimatized to laboratory conditions over a period of two weeks. Six replicate predation trials were carried out for each prey species at four different size classes (mean total length: class 1 = 20.5 mm, class 2 = 26.2 mm, class 3 = 32.1 mm, class 4 = 40.6 mm, total N = 1080). The predatory sticklebacks (mean total length: 683 ± 34 mm, N = 72) were food deprived for 48 h. Before the start of each trial, a randomly selected stickleback was gently placed in one half of a 30 x 30 x 30 cm experimental tank filled with 27 L of clear lake water and divided by an opaque sheet of polyvinyl chloride (PVC). The opposite half of the tank was stocked with N = 15 similar-sized larvae of one prey species. After an acclimatization period of 15 min the PVC sheet was removed. The stickleback was allowed 30 minutes to predate on the larvae. Individual sticklebacks were only used in one trial. Each trial was performed at a water temperature ranging from 18 – 20°C. Illumination was provided by an LED strip (6000 K) mounted on top of the experimental tank, resulting 1600 lux measured in the centre of the experimental tank at 12.5 cm depth. To prevent disturbances during the trial from movements outside the experimental tank, the experimental installation was shielded by optically-opaque curtains.

### Behavioural analysis

**Fig 1:**
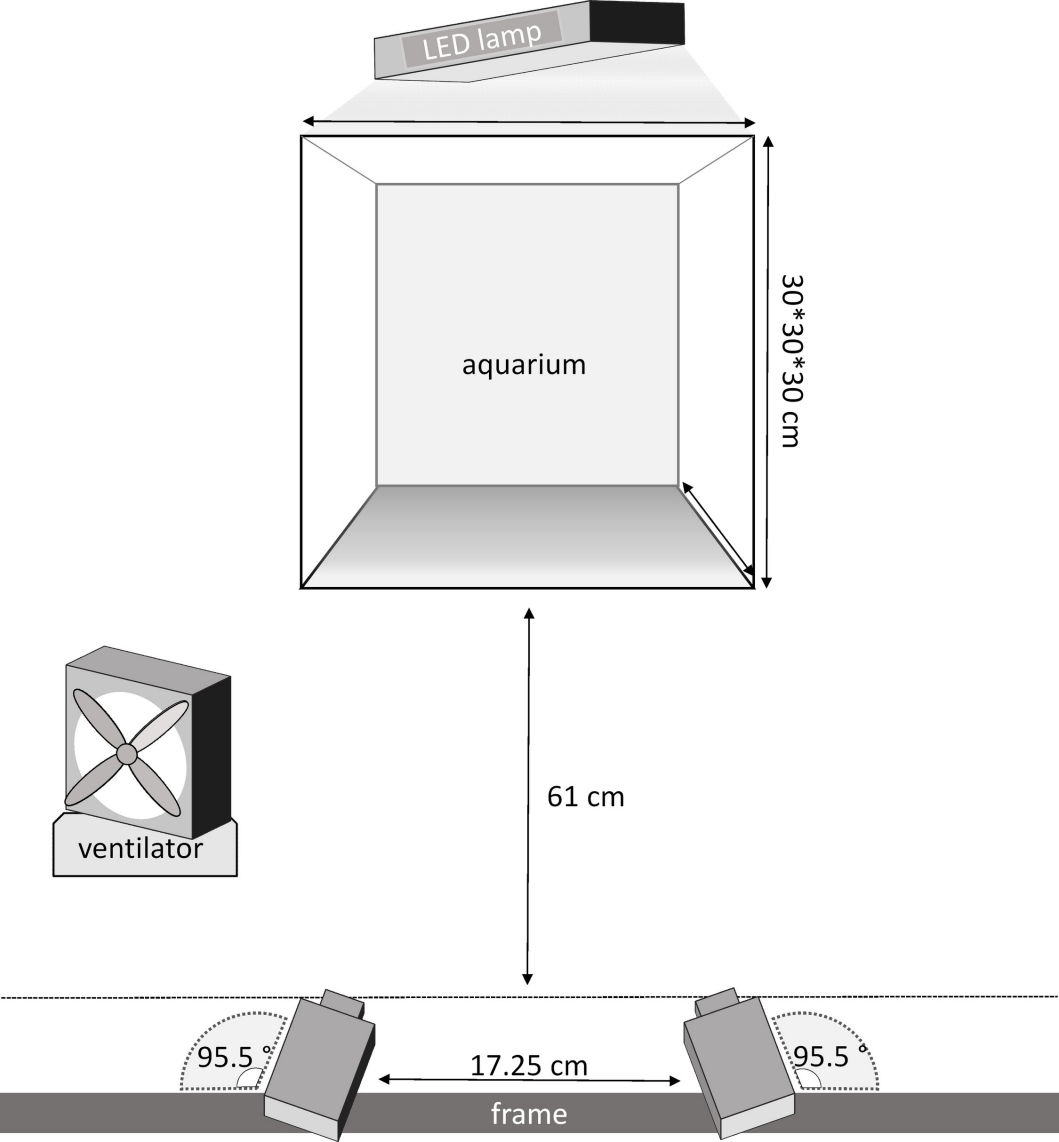
Experimental tank setup. Experimental tank setup consisting of a tank with dimensions of 30 x 30 x 30 cm, two high-speed cameras in a fixed stereo rig, LED lights for illumination and a ventilator to prevent condensation on the glass.

Every predation trial was recorded on video with an accurately synchronized, fixed-frame slow-motion stereo camera rig (Fig 1). Two high frame rate cameras (JAI GO-5000C-USB and Ricoh 12.5 mm objective FL-BC1214D-VG) were used to record the experimental trials at 140 fps with a resolution of 1200 x 900 pixels in MJPG format using the Common Vision Blox Management Console (Stemmer Imaging GmbH, Puchheim, Germany). The program used for video recording was Gecko (GigE VCR, V.2.0.3.1, Vision Experts, Farnham, England). To prevent condensation on the glass, the front of the tank was ventilated with a fan.

The open-source program package Argus:: 3D for the people (V.2.1) [41] was used to calibrate the camera rig to the 3D environment and to manually track the animals. Intrinsic camera parameters, including focal length and optical centre were collected with the Argus-Patterns function. 3D coordinates of the camera were transformed into 2D image coordinates by using a 5 x 7 dot grid pattern and then calculated using the Argus-Calibrate function [44]. The Argus-Wand function was used to calibrate extrinsic parameters, such as the orientation of the cameras in the 3D environment. Extrinsic parameters were used to transform 3D world coordinates to 3D camera coordinates [45]. Furthermore, a wand with two 8 cm spaced dots was recorded simultaneously with both cameras while moving inside the filled aquarium. The dots were tracked with the Argus-Clicker program and imported along with the previously calibrated intrinsic parameters, into Argus-Wand to complete the calibration of the cameras.

To minimise any error that may occur before and during the experiment–for example due to accidental movement of one of the cameras, stereo calibration of the 3D cameras was repeated before every trial. Accuracy of the 3D setup was expected to have an error of < 1%/m [46–49]. To estimate the calibration error, the tracks of the wand (8 cm spaced dots) were analysed, resulting in a mean estimated distance of 8.05 cm (N = 2001, 95% CI ± 0.01 cm). Thus, calibration was accurate with only a slight overestimation of 0.05 ± 0.01 cm.

**Fig 2:**
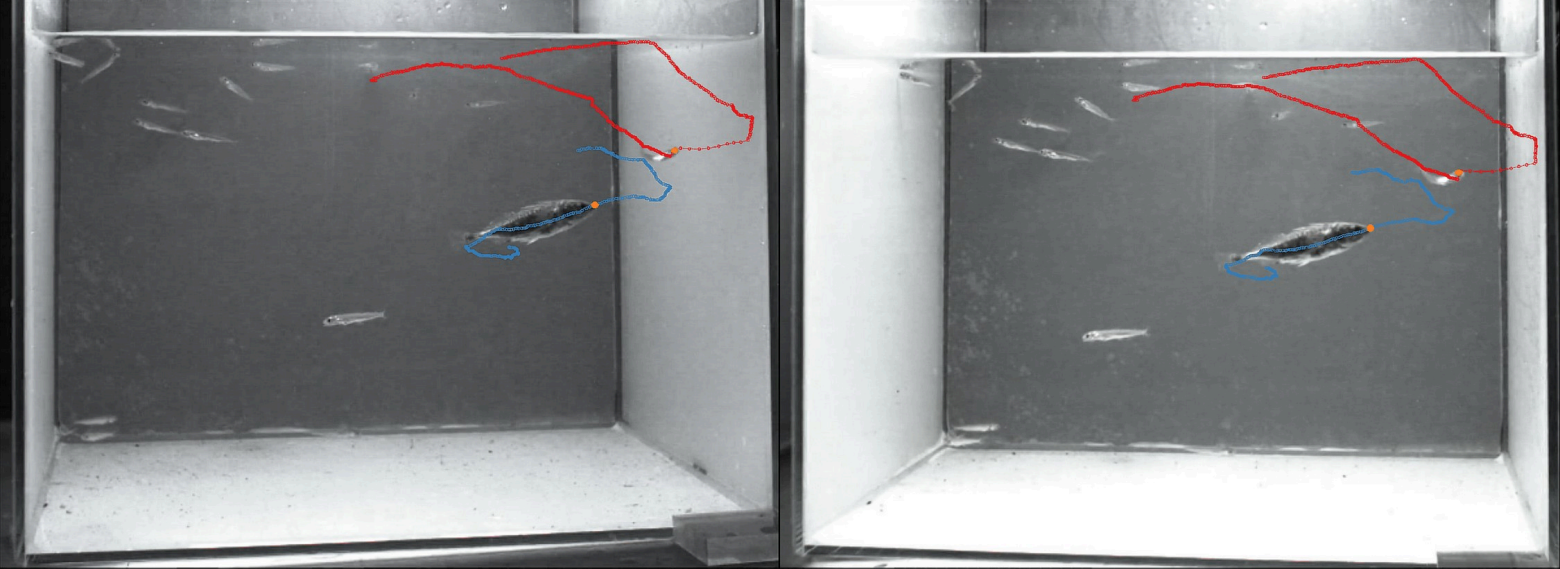
2D stereo tracking. Manual tracking analysis with Argus of left and right camera recording; tracking points: eye of the larvae (red), snout-tip of the stickleback (blue), tracking period: first hunt over 700 frames (5 s). Here the whitefish larva escaped. The setup partially mimics the pelagic situation of Lake Constance.

Each 30-minute video file of a predation trial was first screened visually in order to identify successful and unsuccessful hunts. Hunts in which the predator and prey were not clearly visible for more than 10 consecutive frames were discarded from the analysis and the next possible hunt from the same predation trial was selected. For predator and prey tracking, the first successful and the first unsuccessful hunt were selected to allow for comparative levels during these tracks in motivation or satiation of the stickleback and in predator naivety of the larvae. Every hunt was categorized as one of two outcomes: I) as a successful hunt if any part of the prey was bitten by the stickleback; II) as an unsuccessful hunt when the predator initiated a hunt but did not catch any part of the prey. Missing cases occurred when no stickleback hunting behaviour was observed within the 30 min trial. Manual tracking of the protagonists of each hunt was carried out by the same scientist for all trials, using the Argus-Clicker software (Fig 2). For successful hunts, the animals were tracked from three seconds before the biting event (420 frames). Unsuccessful hunts were tracked from three seconds before the biting attempt, and until two seconds after the attempt (280 frames) to include the successful evasive behaviour of the prey. The tip of the stickleback snout and the eye of the larva were used as track points for predator and prey respectively. These features were selected because they were easily visible and, therefore, allowed for precise tracking on individual frames of the video recordings. If a track point was blocked from camera angle due to orientation or other fish for a maximum of ten consecutive frames, the missing coordinates were estimated by frame-by-frame forward and back tracking. Often the second camera gave visual confirmation of this estimation.

**Fig 3:**
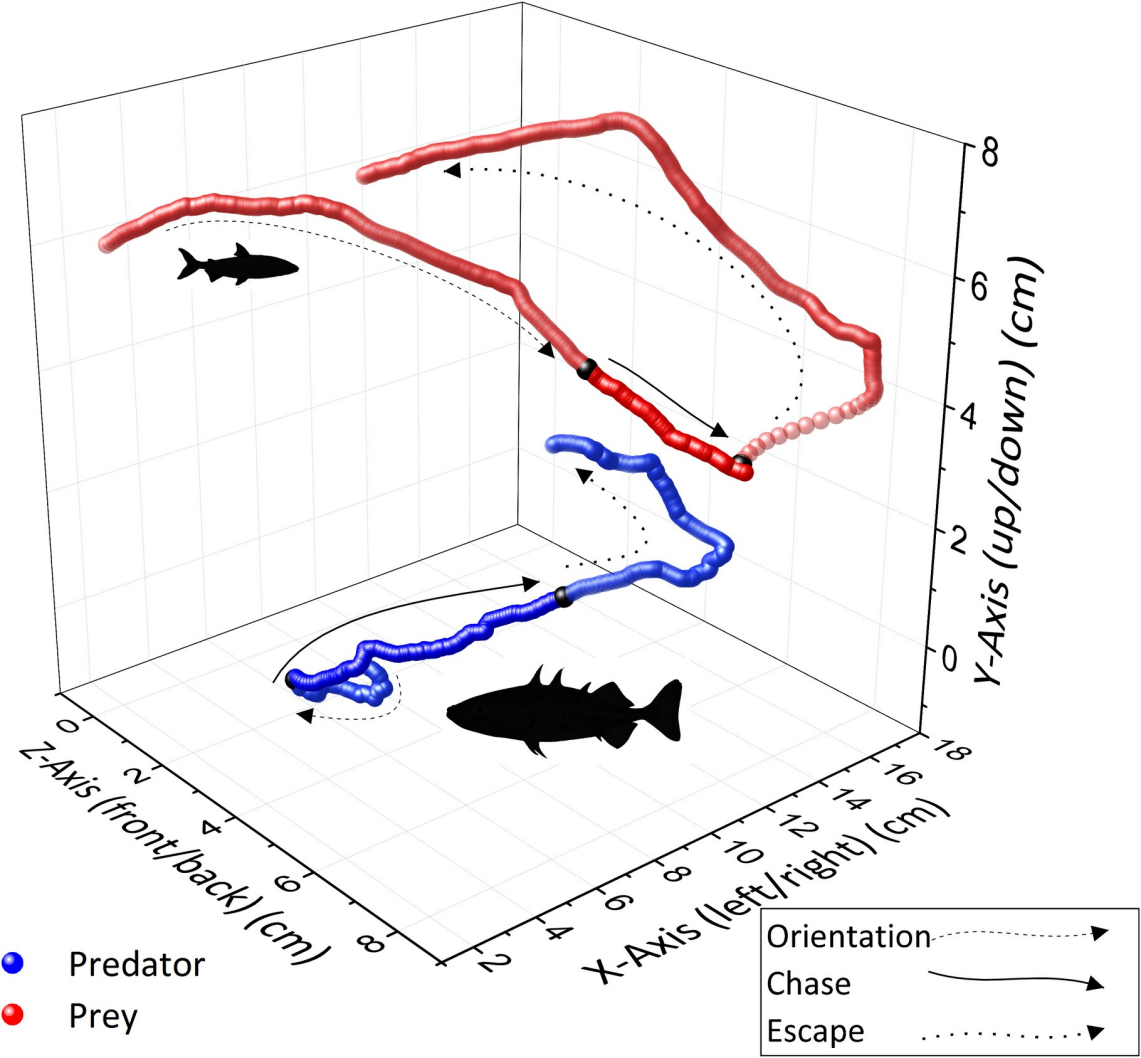
3D analysis. Resulting data points of the 3D analysis were smoothed in Python 3. Depicted are different stages of a failed predation (stickleback: blue, whitefish: red). Tracking started 3 s before capture or escape (400 frames).

For every hunting event, four 2D tracks (two from each camera for predator and prey) were imported into Argus-Wand including the previously estimated camera parameters. The 3D coordinates were calculated in the same program for each individual track. To minimize noise in the data, all 3D coordinates were further smoothed with a code using a running average method (Python v.3.5.4; numpy 1.16) (S1 Appendix) on X and Y coordinates in five preceding and five subsequent frames. For the Z coordinates (depth) a range of ten points was necessary due to larger variance in the estimated positions resulting from calibration. Smoothing resulted in the loss of 10 coordinates (= 0.07 s) at the beginning and at the end of each track. Every track of a successful hunt therefore constituted 260 predator and prey coordinates and an unsuccessful hunt track was made up of 400 coordinates for each protagonist (Fig 3). Within each hunting period, behaviour analysis was carried out from the time the stickleback detected its prey to the time it attempting to bite (set at time = 0 s). The start of the hunt was determined according to two criteria, *i*.*e*., a subjective measure of orientation towards the prey from observation of the video recordings, and a general change in swimming behaviour using breakpoint analysis using segmented linear regression. The second criterion was used to verify the initial observation from the video recordings.

The following formulas were applied to calculate predator-prey interactions and behaviour in python (S2 Appendix):

$$|\vec{PQ}|=\sqrt{{\left(q_{1}-p_{1}\right)}^{2}+{\left(q_{2}-p_{2}\right)}^{2}+{\left(q_{3}-p_{3}\right)}^{2}}$$

(1) Distance between two track points P (p_1_|p_2_|p_3_) and Q (q_1_|q_2_|q_3_) in a three-dimensional space

$$Velocity\;\left(\frac{cm}{s}\right)=\frac{Distance\;(cm)}{Time\;(s)}$$

(2) Swimming velocity of predator and prey

$$Acceleration\;\left(\frac{cm}{s^{2}}\right)=\frac{Change\;in\;Velocity\;\Delta v\;(\frac{cm}{s})}{Change\;in\;Time\;\Delta t\;(s)}$$

(3) Acceleration of predator and prey

$$\vec{PQ}=\left(\begin{array}{c} q_{1}-p_{1}\\ q_{2}-p_{2}\\ q_{3}-p_{3} \end{array}\right)$$

(4) Vector from two track points P (p_1_|p_2_|p_3_) and Q (q_1_|q_2_|q_3_) in a three-dimensional space

$$\cos\;\varphi =\frac{\vec{p}\cdot \vec{q}}{\left|\vec{p}|\cdot |\vec{q}\right|}\;\to \;\varphi =\cos^{-1}\cdot (\frac{\vec{p}\cdot \vec{q}}{\left|\vec{p}|\cdot |\vec{q}\right|})$$

(5) Angle between two vectors $\vec{p}$ and $\vec{q}$ in a three-dimensional space

### Statistical analyses

In order to assess the point in the hunt where predator and prey adjusted their behaviour for the first time, a breakpoint analysis was conducted on the respective datasets for successful and failed hunts. For the hunter, the breakpoint may indicate the end of the orientation phase and the initiation of the hunt. For the prey, the breakpoint may signify the detection of the predator and the initiation of flight. The breakpoint analysis was carried out in R (version 3.6). The optimize algorithm (package stats) was applied to a segmented linear regression model with subject as random effect (using lmer from package lme4) in order to select the breakpoint with lowest deviance, and estimate its variance. Speed of the predator or prey was chosen as a dependent variable, and square root transformed to meet the normality assumption. In order to assess statistical differences between groups, t-values were calculated by means of the difference in breakpoints, the pooled variation between the groups, and the number of trials on which comparisons were based.

Further statistical analysis of the data was conducted with the software JMP Pro (version 14.3.0 64-bit, SAS Institute, Cary, North Carolina, USA). Figures were created in Origin 2017 (version b9.4.2.380, 64-bit, OriginLab Corporation, Northampton, Massachusetts, USA). The relationships between hunting success (*i*.*e*. successful *vs*. failed hunt) and predator-prey performance variables, such as swimming velocity (cm/s), acceleration (cm/s^2^) and turning angle (°), were tested using univariate logistic models. Predation trial was entered as random effect in the statistical models. All variables are reported as mean ± standard deviation. In order to further explain variation in hunting success over all species and size classes, the above-mentioned performance variables (average swimming velocity (cm/s), average acceleration (cm/s^2^) and average turning angle (°), time before attack (s), predator-prey distance start (cm) and minimal predator-prey distance (cm)) were entered in a multivariate logistic regression along with interactions between species and prey-size (see variables S2 Table). The best-fit variables were selected through forward and backward stepwise selection (selection criteria: enter variable when *P <* 0.05, leave variable when *P >* 0.07).

### Ethics statement

All trials were conducted according to the German Animal Welfare Act (TierSchG), under an ethical permit granted by the Regierungspraesidium Tuebingen, Referat Tierschutz (LAZ 1/17).

Approval of the collection of animals for the present study by a review board institution or ethics committee was not necessary because all fish were caught under the permission of the local fisheries administration (Regierungspräsidium Tübingen) and all needed qualifications for the involved people (fishing licenses) were checked regularly by the local fisheries administration (Regierungspräsidium Tübingen). All fish were caught according to the German Animal Protection Law (§ 4) and the ordinance on slaughter and killing of animals (Tierschutzschlachtverordnung § 13). The present study did not involve endangered or protected species.

## Results

### General description of hunting tracks

In total, 75 predator-prey interactions were analysed, including 24 failed and 20 successful hunts for whitefish, 17 failed and 4 successful hunts for roach and 9 failed hunts and 1 successful hunt for perch. Hunts involving perch and roach were pooled together in the statistical models and results, were necessary (in Fig 5 and the multivariate logistic regression), as these show similar anti-predator behaviour clearly different from that of whitefish (schooling, zigzagging) [33] and to increase the N-value in this group. To evaluate the speed of the prey, an additional 18 tracks of failed and incomplete hunts were analysed from size classes where no successful hunts took place (perch size class 3 and 4; roach size class 4) (S1 Table). For further details of stickleback hunting success and variation in predator avoidance strategies shown by prey in this experiment see Ros *et al*. [33].

**Fig 4:**
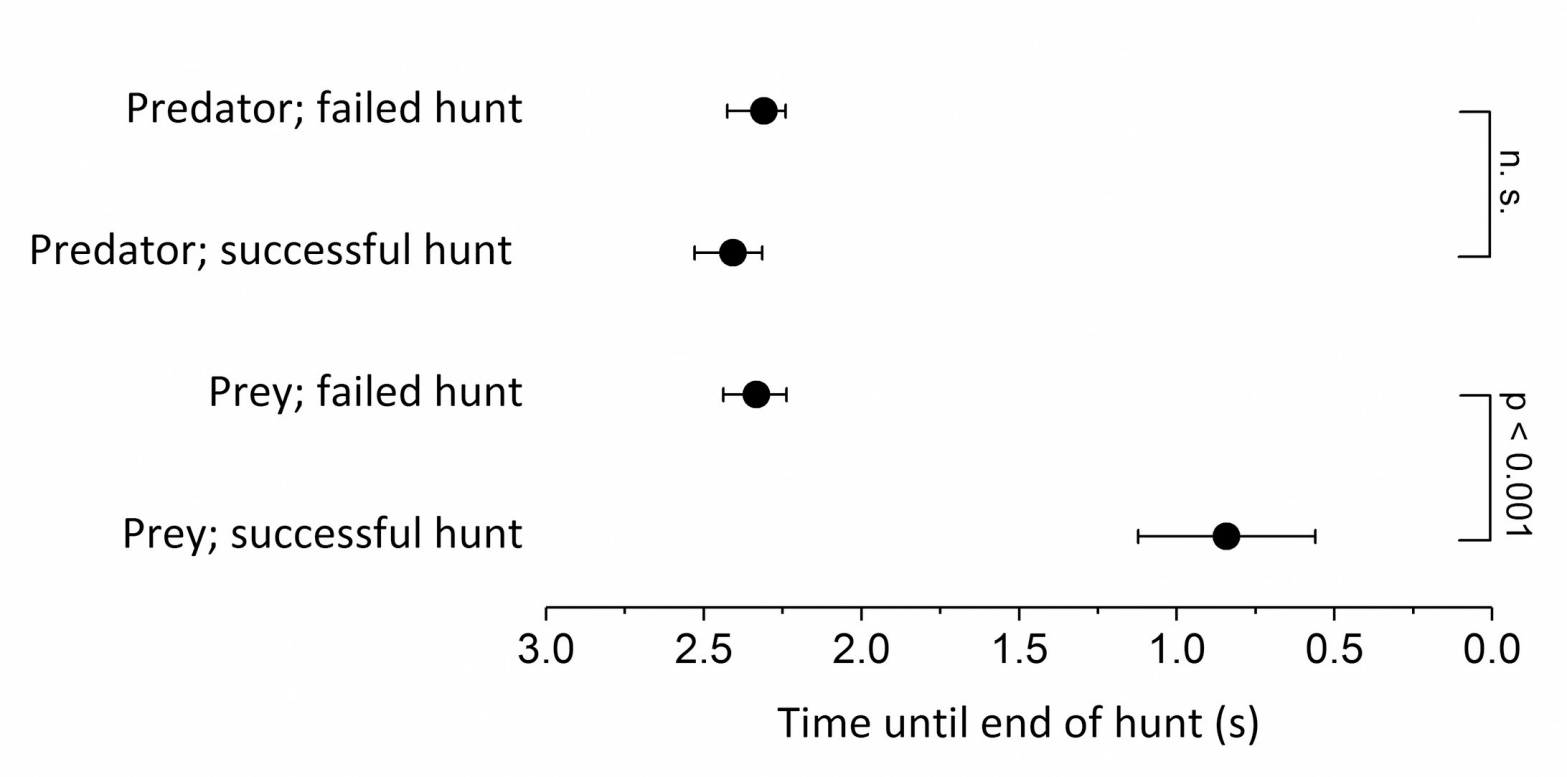
Breakpoint analysis. To identify changes in swimming speed in successful and unsuccessful whitefish hunts, the first breakpoint was selected using segmented linear regression analysis of the three second period before the biting event or attempt.

At three seconds before the biting attempt, predators did not yet show any clear targeted swimming towards the prey (orientation). The first indication of hunting behaviour was a change in the swimming speed of the predators just less than 2.5 seconds before the biting attempt (Fig 4). Whitefish larvae in a successful hunt showed a significantly delayed response to hunting behaviour of the stickleback (t-test: *P <* .001 [t_22_ = 9.8, N = 44]).

**Fig 5:**
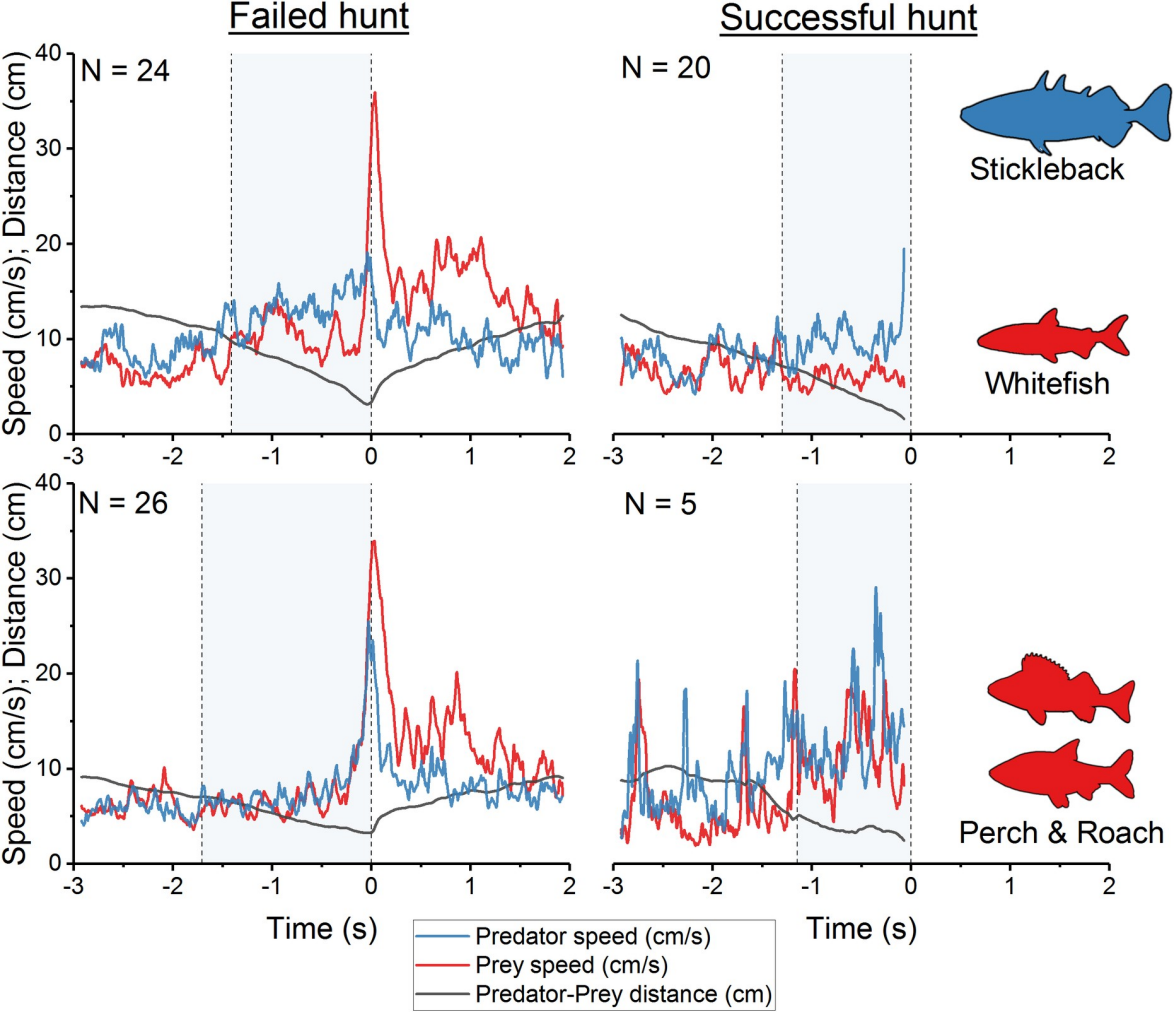
Predator and prey tracks before and after an unsuccessful hunt and before a successful hunt. The biting attempt of the predator was at time = 0 s. Tracks were divided into two prey fish categories (whitefish, and roach & perch). The speed in cm/s and distance in cm was derived from smoothed 3D tracks of prey and predators. Tracks were averaged over each species and outcome category. The grey-coloured period of predation describes the hunt of the stickleback.

At this time, the predator changed its orientation towards the prey while still swimming at a low speed of 8.11 ± 7.7 cm/s. Then, during the hunt, the sticklebacks closed in on their potential prey while accelerating slightly (Fig 5).

During orientation towards the prey, the average initial distance between protagonists was 7.6 ± 5.1 cm. 3D tracks showed that from the first moment of directed swimming to the moment of prey capture or failed predation, average hunt duration was 1.4 ± 0.8 seconds. During this directed hunt the predators spanned an average distance of 15.9 ± 10.7 cm and the prey travelled on average 13.4 ± 13.1 cm, displaying an average speed of 11.5 ± 4.8 cm/s and 10.4 ± 6.3 cm/s, respectively. The minimal distance between predator and prey calculated from the last frame after which the prey was either captured or managed to escape was 1.7 ± 1.2 cm (Table 1).

**Table 1:** Performance data of predator and prey fish during the hunt.

N = 75; N = 93†	mean ± SD
timespan of hunt (s)	1.4 ± 0.8
initial distance (cm)	7.6 ± 5.1
minimal distance (cm)	1.7 ± 1.2
distance travelled prey (cm)	13.4 ± 13.1
distance travelled predator (cm)	15.9 ± 10.7
speed prey (cm/s)†	10.4 ± 6.3
speed predator (cm/s)	11.5 ± 4.8
initial speed predator (cm/s)	8.1 ± 7.7

Generalized overview of performance data of predator and prey fish during the hunt across all species and outcome, 75 predator prey interactions were observed to identify performance of predator and prey. To evaluate the speed of the prey, additionally 18 tracks of fast swimming larvae (marked with †) were included from size classes where no successful hunt took place (perch size class 3 and 4; roach size class 4).

### Hunting different prey species

Successful hunts of the different prey species generally followed a similar pattern, although the tracks were more variable in hunts of perch and roach than in those of whitefish (CV = 58.5 *vs*. 21.6; Fig 5). Also, in both failed and successful whitefish hunts, the acceleration of the predator as it closed on its prey was largely independent of prey speed. However, in a clear difference from successful hunts, whitefish, which escaped, accelerated their swimming speed in the first quarter of failed hunts (Fig 5). This was detected in the breakpoint analysis as a significant difference in the timing of the change in swimming speeds of whitefish larvae between successful and unsuccessful hunts (Fig 4, t-test: *P <* 0.001 [t_22_ = 9.8, N = 44]). Failed whitefish hunts were characterized by an initially slow approach of the predator, followed by rapid acceleration in the last tenth of a second before the end of the hunt. In contrast, the swimming speeds of perch and roach larvae indicated a more reactive pattern in which the acceleration of the predator was effectively mirrored by acceleration of the prey (Fig 5).

Maximum recorded speeds (cm/s) were significantly different between prey species (*P =* 0.04 [F_2,62.4_ = 3.38; N = 75], S3 Table). The measure in whitefish larvae (45.8±19.1 cm/s) was intermediate between roach (56.9 ± 28.8 cm/s) and perch (39.3±18.4 cm/s) (S2 Table). The highest performance was found for roach, which reached 45% greater top speeds than perch (S2 and S3 Tables), although both species were successful in escaping from the predator. The average turning angle of the prey larvae did not differ significantly between species (roach: 13.0 ± 5.5, whitefish: 14.3 ± 5.3, perch: 15.0 ± 5.1; S3 Table).

Both the maximum speed (*P =* 0.024 [F_2,44.7_ = 4.07; N = 50], S3 Table) and the average turning angle of stickleback predators (*P =* 0.03 [F_3,41.6_ = 3.28; N = 50], S3 Table) varied significantly depending on the prey species. Escaping perch larvae lead to significantly (28%) larger turning angles in the predator than escaping roach or whitefish, while escaping roach larvae induced the fastest swimming by stickleback (roach: 50.7 ± 21.2 cm/s, perch: 31.9 ± 15.0 cm/s S2 and S3 Tables). Again, the whitefish larvae were intermediate performers in this variation (36.2 ± 16.5 cm/s).

### Hunting larvae of different size classes

**Fig 6:**
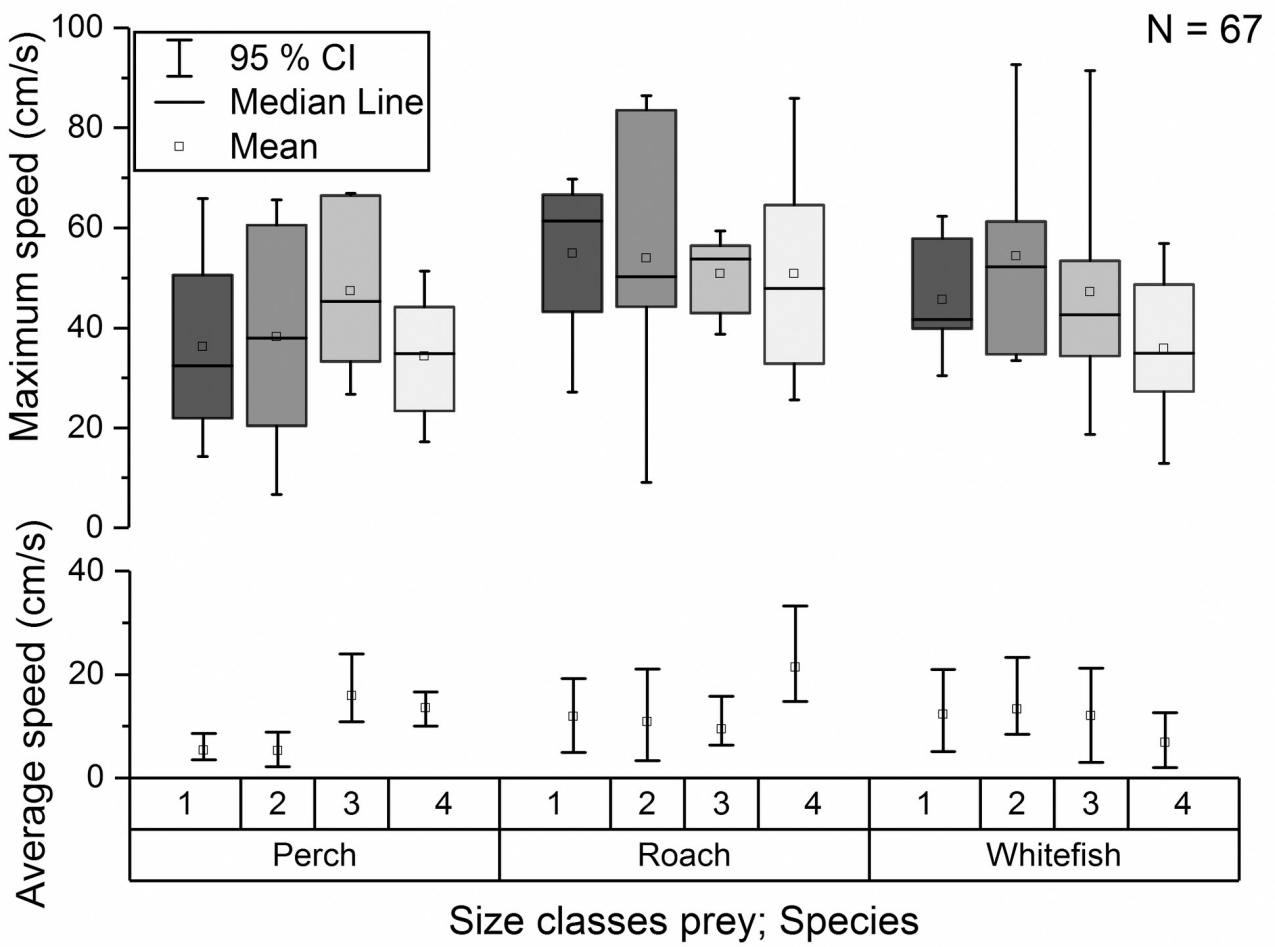
Maximum and average speed of prey. Maximum and average speed of prey (cm/s) in failed hunts divided by species and size class.

**Fig 7:**
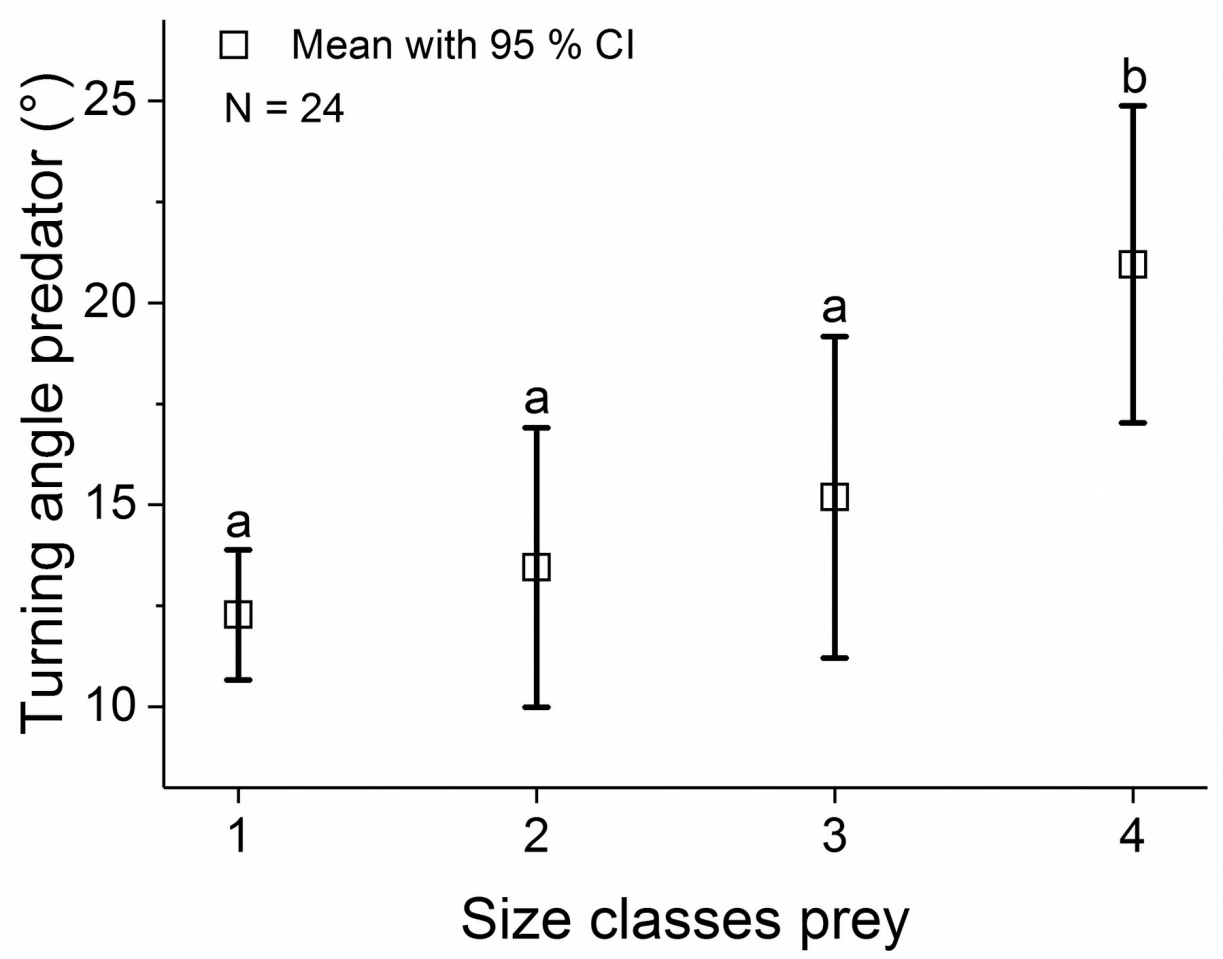
Average turning angle of the predator. Average turning angle of the predator during the failed hunts of whitefish over the four size classes of prey.

None of the performance measurements of whitefish showed any significant increase with larval size (S5 and S7 Tables). For roach and perch only the average speed and the average turning angle of the prey differed between the size classes (speed: *P =* 0.014 [F_3,530.9_ = 4.64; N = 24] and *P <* 0.001 [F_3,471.2_ = 11.00; N = 22], angle: *P =* 0.034 [F_3,236.3_ = 3.56; N = 24] and *P =* 0.001 [F_3,314.8_ = 8.56; N = 22]; Fig 6, S7 Table). Furthermore, most performance variables describing the behaviour of the predator did not change statistically with the size of the larvae (S7 Table), with the exception of the variable turning angle. This variable revealed increasingly abrupt changes in swimming direction of the predator when hunting whitefish larvae of increasing size (Fig 7, *P =* 0.001 [F_3,266.2_ = 8.55; N = 24], S7 Table). The predator displayed an average turning angle of 12.3 ± 1.5° for hunting the smallest class of whitefish, while this increased to 20.9 ± 3.7° when hunting the largest size class.

### Factors affecting a successful hunt

Comparison of successful and failed hunts revealed several performance differences between prey species (S2 Table). Prey escaping from predation exhibited significantly faster acceleration (*P =* 0.02 [F_3,12.4_ = 4.73; N = 75], S3 Table) and reached significantly higher maximum speeds than prey captured by the predator (roach: 50.7 *vs*. 40.2 cm/s, whitefish: 36.2 *vs*. 27.7 cm/s, (*P <* 0.001 [F_3,52.0_ = 7.76; N = 93], S2 and S3 Tables). In whitefish, the maximum speed was lower than that of roach (see above), but mean speed was also higher in escaping whitefish than captured ones (*P =* 0.042 [F_1,21.3_ = 4.67; N = 44], S3 Table) but not in roach (*P =* 0.629 [F_1,14.1_ = 0.24; N = 27], S3 Table).

The performance of the stickleback predators in successful and failed hunts varied with prey species between whitefish and roach. In hunts for roach, highest average swimming speeds were found in sticklebacks that were successful in catching the prey (*P =* 0.03 [F_1,20.8_ = 5.43; N = 21], S3 Table), but the opposite was found in whitefish (*P =* 0.004 [F_2,34.3_ = 6.35; N = 44], S3 Table). Overall, the highest maximum predator accelerations were recorded in hunts which ultimately failed (S2 Table).

A multivariate logistic regression revealed how performance variables combine to determine the success or failure of a hunt from the perspective of the stickleback over all prey species and size classes (Table 2).

**Table 2:** Variables predicting the successful outcome of the predation from a predator’s perspective.

	estimate	standard error	χ2	*P*
intercept	-6.953	2.152	10.441	0.001
species [perch&roach *vs*. whitefish]	-0.898	0.519	2.993	0.084
avg. speed prey (cm/s)	-2.793	0.861	10.516	0.001
avg. acceleration prey (cm/s^2^)	-11.436	3.65	9.814	0.002

**Fig 8:**
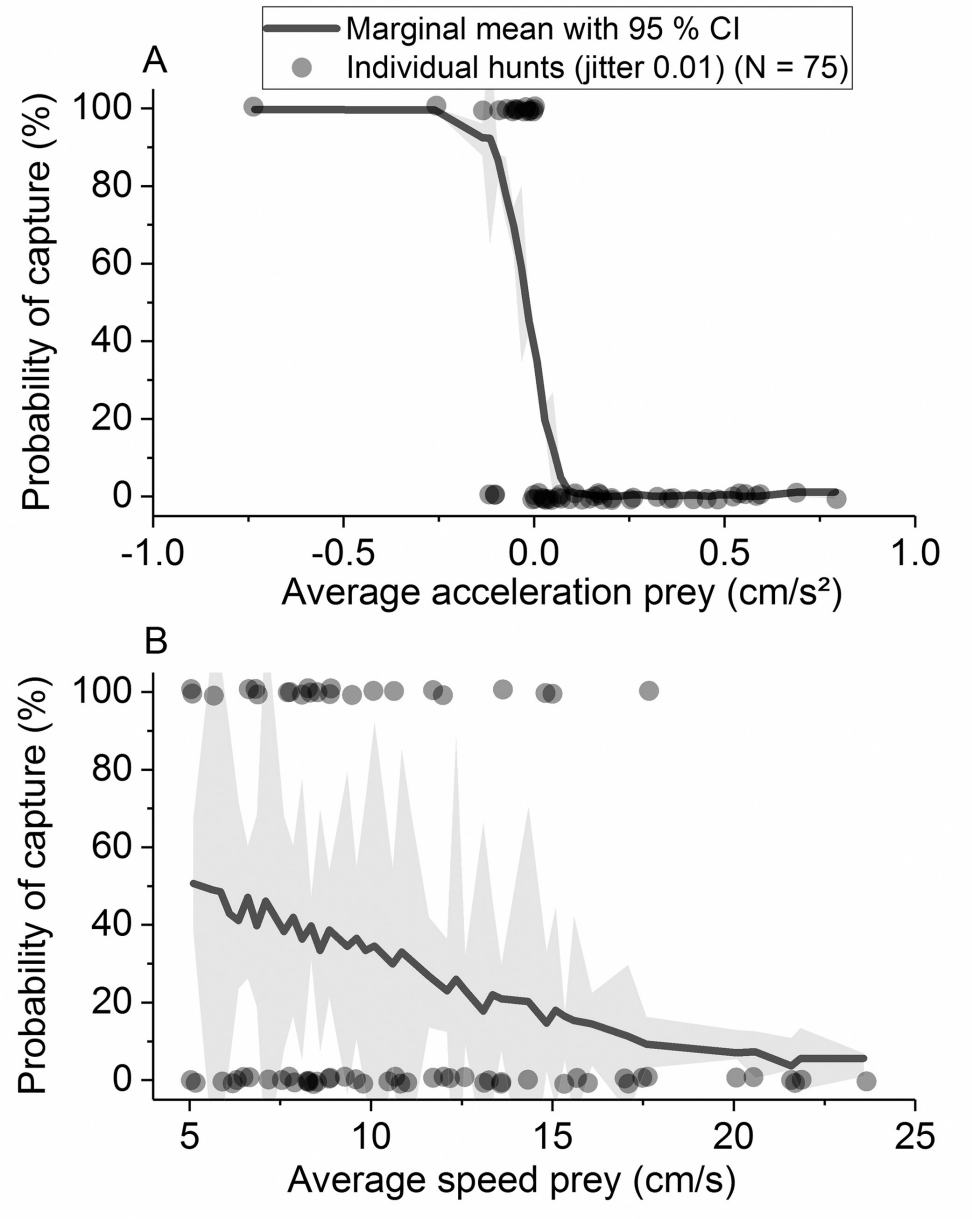
Likelihood to capture prey. Output of the multivariate logistic regression depicted the likelihood of the predator to capture the prey in relation to A) the average acceleration of the prey and B) the average speed of the prey, N = 75 predation events, including all prey species and outcomes.

Both factors predicting the outcome were related to the performance of prey fish. A low average speed and a slow acceleration of prey resulted in a higher possibility of stickleback success (Fig 8). These results are also visualised in the generalized tracks of Fig 5 and the delayed response of the prey in Fig 4.

## Discussion

A detailed 3D-analysis of the interaction between stickleback predators and larval fish prey showed a strong influence of the latency in the hunting-induced escape response on hunting success. Hunting was mostly unsuccessful when prey showed early awareness of the predator and began to accelerate when the predator was still more than 5 cm away. This pattern was most clearly seen in hunts of smaller size-classes of whitefish larvae, which exhibit relatively limited predator avoidance behaviour as previously shown in the experiments [33]. In contrast with whitefish, roach and perch larvae possess more elaborate flight responses (see exemplarily S1–S3 Figs). Sticklebacks approached these larvae at generally higher speeds inciting a stronger reaction from the larvae in terms of swimming speed. In general, sticklebacks tended to approach their prey slowly, using relatively simple straight tracks, before accelerating in the last tenth of a second before snapping at the prey. Despite differences between prey species in swimming performance variables like maximum speed, these did not explain the differences in escape success of the larvae. The slowest (perch) and the fastest (roach) species had high escape success, whilst the species in between (whitefish) had a low escape success. Both species and size exerted an effect on the turning angles recorded in the hunting tracks of the predator. This was reflected in the more variable tracks of sticklebacks when hunting large whitefish larvae compared to small ones and when hunting perch rather than whitefish. It indicates that sticklebacks had to make larger adjustments when pursuing larger prey, resulting in a lower success rate.

### Stickleback hunting strategies on fish larvae

The 3D track analyses of predator-prey paths show that sticklebacks typically executed brief, stealthy approaches, oriented directly towards the prey, culminating in a rapid strike from close range, as has been found in other teleost and mammalian predators [50–53]. The observed direct pursuit of prey indicates that sticklebacks use a constant bearing strategy in which the predator tries to keep the prey in the “line of sight” [54] (see Fig 3). The available literature on predation behaviour indicates that this is a typical strategy among fish. In zebrafish, for example, hunts are carried out using a pure constant bearing (*Danio rerio*) [55], while bluefish (*Pomatomus saltatrix*) [56], and leather jacket (*Acanthaluteres spilomelanuru*) exhibit a slight variation of this strategy in which prey is approached from below but not from above [57]. A possible benefit of direct pursuit might be that the length dimension of the predator remains obscured from the prey, making accurate estimation of the size of the approaching fish more difficult and, thereby, delaying its flight response. Also consistent with direct pursuit is that the trajectories of predator and prey tracks show a nearly linear decline in distance over time. In case of unsuccessful hunts, there were no differences in predator and prey speed, so the track lengths achieved were similar for predator and prey (17.7 cm and 17.6 cm resp.). Although track lengths were similar, the predators closed in on the prey by increasing speed over a distance of 5.6 cm. This result means that sticklebacks tended to select larvae that were swimming perpendicular or even slightly towards the predator.

The rapid decline in speed of the sticklebacks following an unsuccessful strike at the prey may indicate that they had difficulty altering their trajectory once they commenced their hunt and accelerated towards their prey, and that after being outmanoeuvred they tend to lose their bearing on the prey and abort the hunt. Related to this, maximum speeds of the predators were clearly lower than those of the prey. Previous studies have reported burst speeds in adult sticklebacks ranging from 60 cm/s to 100–130 cm/s [58–60], substantially higher than the maximum speed of sticklebacks in failed hunts recorded in this study (40.4 ± 19.2 cm/s). These differences in maximum swimming speed can be explained by the fact that the current experiment measures voluntary foraging tracks, whereas previous studies elicited escape responses using mechanical or artificial input. Furthermore, burst speeds of predators seen during the hunt are likely to be lower than the maximum speed that predators can achieve, because they have to remain with sufficient resources to be able to successfully perform in future hunts and ultimately to ensure a positive energy balance from feeding. Moreover, prey investing primarily in burst speed to avoid predation typically divert from the track of the predator [42] and therefore, the predator must balance its speed against agility: responding to prey manoeuvres may only be possible at lower than maximum speeds. A trade-off between speed and agility has been shown in terrestrial pursuits [61], but previous observations on fishes suggest that increased speed does not necessarily affected manoeuvrability [62]. Finally, as an opportunist predator, the stickleback is likely to encounter multiple opportunities in a feeding period and this decreases the importance of any single predation attempt [53].

### Differences between failed and successful hunts

The multivariate analysis indicated that larval acceleration rates and swimming speeds were key factors affecting the hunting success of sticklebacks in the current trials, with faster acceleration and high speeds significantly increasing the likelihood of hunting failure. Comparison of successful and failed hunts shows a significant divergence between the tracks of captured and escaped prey, which becomes apparent within the first second of the hunt (Fig 4). This was most evident in whitefish larvae, in which, individuals that did not accelerate successfully tended to be predated, whereas those that responded early in the hunt generally managed to escape. On account of their large scales, which serve as defensive armour plating, sticklebacks have a relatively rigid body which limits their ability to accelerate fast, and may be an extra handicap when responding to the evasive strategies of highly manoeuvrable prey [24, 63, 64]. However, since all the predators in the experimental setup were plated, observed variations in hunting success are more likely to be linked to prey performance, with larvae able to achieve a faster response being more likely to outperform or outmanoeuvre their large stickleback predator.

Previous analyses of stickleback predator-prey interactions indicated that prey species differ in escape success through species-specific variations in predator avoidance behaviour. Whitefish larvae favour direct flight [33, 65], while perch and roach larvae exhibited more elaborate predator avoidance responses, like schooling and zigzagging behaviour [33]. This explains the observed lower variation in predation tracks (in terms of change of direction) observed in sticklebacks hunting whitefish than those hunting perch. It further indicates that the strategy deployed by the three-spined stickleback to intercept its prey is more successful when larvae use predictable escape responses, i.e., direct flight, than when they use more elaborate behaviour.

### Hunting of increasing sizes of prey

**Fig 9:**
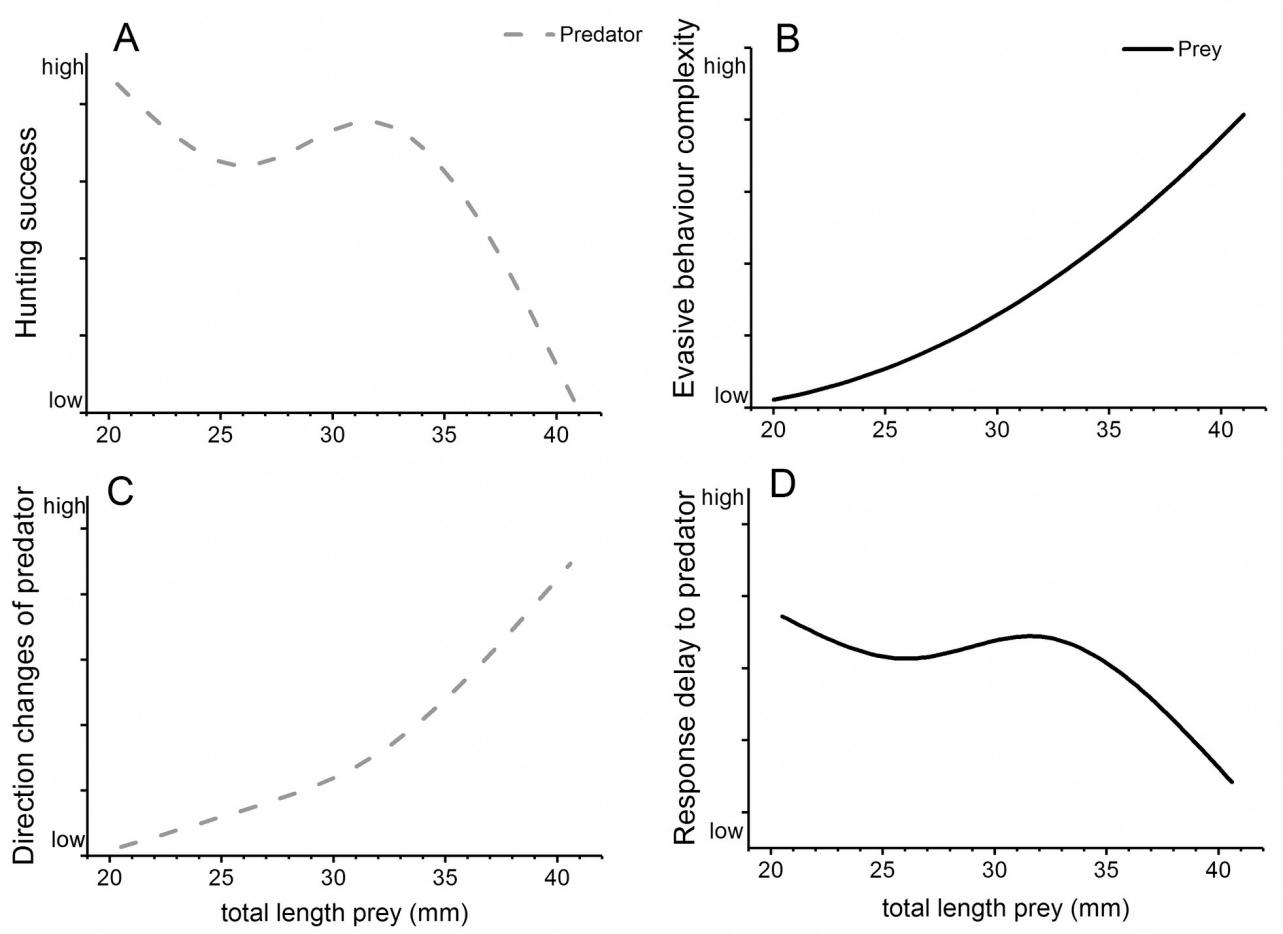
Projected predator and prey relationships. Summary of the observed relationships between predator (stickleback) and prey (whitefish) behaviour at different prey length classes: A) Hunting success of sticklebacks decreases with prey size, which was shown to be correlated to B) an increase in complexity of predator avoidance behaviours. A and B are based on previously published data from the same experiment [33]. The current study indicates that: C) predators more often adjust their swimming direction when following larger whitefish larvae; D) larger whitefish are increasingly adept at detecting the start of the hunt as measured by an increase in swimming speed; B+D could explain why predators increasingly need to adjust their track C) which in turn might decrease hunting performance (A).

Sticklebacks hunting whitefish larvae which lacked previous predator experience are less successful with increasing size of the prey [32]. In a published paper based on the current experiment this prey size effect was confirmed [33], and it was postulated that the most parsimonious explanation for this effect was an increase in performance variables of the prey with increasing size, like in maximum acceleration and in swimming speed [66, 67]. However, in the current 3D analysis of this study, the acceleration rates and maximum speeds achieved by smaller larvae were found not to be significantly lower than those of larger larvae. Stickleback adjusted the speed during the hunt, appearing capable to some extent of matching their hunting behaviour to feedback from the prey individual. Therefore, the explanation for the declining predation success with increasing prey sizes must lie in increasingly diverse and complex predator avoidance responses during whitefish ontogeny [33] (Fig 9A and 9B). Related to this change in prey behaviour, our analysis of predator tracks shows an increasing change in the orientation of predators hunting larger whitefish larvae (Fig 9C). Apparently, only small whitefish larvae were using tracks that could be intercepted by a straight pursuit. As argued above related to differences between species, this difference could be due either to sudden changes in the trajectory of the prey, or to prey beginning to exhibit schooling behaviour in which it becomes more difficult for the predator to focus on a single individual. Finally, the largest whitefish group responded the fastest to a perceived threat (Fig 9D). When initiating a hunt, sticklebacks changed speed at much the same time regardless of the size class of the larvae.

### Physical and physiological limitations

A possible reason for the consistency of prey performance variables is that over the investigated size range of 10–40 mm, larvae are differentially affected by forces in physical environment that oppose their movements. At increasing Reynolds values with larger sizes, the larvae experience less friction from water viscosity, but have to deal with increasing drag from water pressure and acceleration reaction [68]. Accordingly, some evidence exists that acceleration and maximum speeds do not increase with length within the size range studied here [63]. In the current setup, sticklebacks did not engage in prolonged hunts. Other benefits and costs of larger bodies to consider are that, on the one hand, larger fish might have greater endurance and swim for longer at maximum speed, while, on the other hand, they are easier for predators to detect and to follow.

Finally, an explanation for the declining trend in certain performance parameters of the prey could be the increase in awareness of larvae of all species. It has previously been shown that the anti-predator response to a predator of fixed size decreases with the size of the prey [69]. It may be that certain sensory capacities which are only triggered after a specific developmental stage, might make larvae better able to assess the threat from a predator and thereby to respond with a more adequate anti-predator mechanism, even without learning. In the case of whitefish larvae, the development of schooling behaviour seems to allow larvae to reduce the effort required in terms of speed in order to successfully escape the predator.

### Ecological impact

Whitefish larvae smaller than 4 cm are shown to be prone to predation by sticklebacks, due to their delayed predator response and lack of adequate anti-predator behaviour. In Lake Constance, whitefish of these size classes typically thought to occur in the pelagic zone, where predators were previously considered to be scarce [34]. However, a new spatial and temporal overlap resulting from an increased abundance of sticklebacks in this part of the ecosystem may result in a high mortality of whitefish larvae and significantly impact the strength of the young of the year class of whitefish during this stage of ontogeny. In whitefish over and above 4 cm, ontogenetic changes of behaviour may help to reduce the predation risk posed by sticklebacks.

## Conclusion

This study underlines predation as a dynamic interplay between behavioural strategies exhibited by predators and prey. It indicates that sticklebacks engage in short-duration hunts, in which they deploy a direct bearing strategy. Within whitefish different strategies to avoid predation were detected. Only the smallest size classes (20.5–32.1 mm) primarily relied on simple direct flight responses, which were easily followed by the predator. Measurements of maximum speed and acceleration indicate that whitefish larvae across the size ranges tested were able to achieve similar performance, but that smaller larvae were more vulnerable to predation than larger larvae. A main difference in hunting tracks between escaping and captured larvae was in the timing of acceleration and the speed that was reached just before the final lunge of the predator towards the prey. The later the larvae accelerated, the greater the chance of success for the hunter. A comparison of prey species showed that more complex evasive behaviours resulted in more adjustments in the hunting track of the predator. Since none of the larvae in our trials had any prior expose to predators, the results reveal possible explanations behind the previously depicted variation in vulnerability between species and between individuals at different developmental stages [33]. Different exposure to predation in the natural habitat of these different species may have shaped this variation. Thus, it appears that appropriate anti-predator behaviour is not present in the earliest life stages of all fish. The anti-predator mechanisms used by some species only develop at a particular size threshold, making smaller individuals especially vulnerable to changes in their ecosystem.

## Supporting information

S1 Appendix Python code (opened in jupyter notebook (5.1.0rc1)) used for smoothing three-dimensional coordinates of tracked animals. (DOCX)

S2 Appendix Python code (opened in jupyter notebook (5.1.0rc1)) used for calculating performance data from three-dimensional coordinates of tracked animals. (DOCX)

S1 Fig 3D-Tracks of a failed hunt between a perch larva (red) and a stickleback (blue). The black triangles mark the start of both tracks, while the black circles mark the end.
(DOCX)

S2 Fig 3D-Tracks of a failed hunt between a roach larva (red) and a stickleback (blue). The black triangles mark the start of both tracks, while the black circles mark the end.
(DOCX)

S3 Fig 3D-Tracks of a failed hunt between a whitefish larva (red) and a stickleback (blue). The black triangles mark the start of both tracks, while the black circles mark the end.
(DOCX)

S1 Table Number of evaluated failed and successful predator-prey interactions per prey species and size class. (DOCX)

S2 Table Performance characteristics of predator and prey stratified by hunting outcome and prey species. The (†) below the species marks, what N was used for the corresponding performance variable per column.
(DOCX)

S3 Table Statistical outcome of univariate logistic models of performance variables (left to right): Roach, comparison between failed and successful hunts, corrected for trial (predator subject) and larvae size class (random effect); whitefish, comparison between failed and successful hunts, corrected for trial and size class; comparison between species within failed predations, corrected for size class; overall failed and successful hunts, corrected for predator and size (species nested). (DOCX)

S4 Table Performance characteristics of sticklebacks and perch, stratified by size class of perch as prey in the failed predation trials. The (†) below the size marks, what N was used for the corresponding performance variable per column.
(DOCX)

S5 Table Performance characteristics of sticklebacks and whitefish, stratified by size class of whitefish as prey in the failed predation trials. (DOCX)

S6 Table Performance characteristics of sticklebacks and roach, stratified by size class of roach as prey in the failed predation trials. The (†) below the size marks, what N was used for the corresponding performance variable per column.
(DOCX)

S7 Table Outcome of the statistical comparison of the effect of size class on performance characteristics stratified for the different prey fish species. Only failed hunts were considered due to low n-values of successful hunts of perch and roach.
(DOCX)
