# Supplementary material for: The hunter and the hunted—A 3D analysis of predator-prey interactions between three-spined sticklebacks (Gasterosteus aculeatus) and larvae of different prey fishes
Source: PLoS One. 2021 Aug 26;16(8):e0256427. doi: 10.1371/journal.pone.0256427 (PMC8389440; doi:10.1371/journal.pone.0256427)
Supplement: S1 Appendix — (DOCX) [file pone.0256427.s001.docx]

**S1 Appendix. Python code (opened in jupyter notebook (5.1.0rc1)) used for smoothing three-dimensional coordinates of tracked animals.**

#the relevant python packages are imported

%matplotlib notebook

import pandas as pd

import numpy as np

#read .csv file and drop cells without value

data = pd.read_csv('file.csv', sep=',', decimal=".")

data = data.dropna()

#define data as arrays

x_1 = np.asarray(data.x_1)

y_1 = np.asarray(data.y_1)

z_1 = np.asarray(data.z_1)

x_2 = np.asarray(data.x_2)

y_2 = np.asarray(data.y_2)

z_2 = np.asarray(data.z_2)

#define smoothing function

def smooth(track, box_pts):

box = np.ones(box_pts)/box_pts

track_smooth = np.convolve(track, box, mode='same')

return track_smooth

#save smoothed data as .csv file

np.savetxt('export_file.csv', np.c_[smooth(x_1,5)[10:-10],smooth(y_1,5)[10:-10],smooth(z_1,10)[10:-10],smooth(x_2,5)[10:-10],smooth(y_2,5)[10:-10],smooth(z_2,10)[10:-10]], fmt='%10.5f', delimiter=',', header='X_1-Axis,Y_1-Axis,Z_1-Axis,X_2-Axis,Y_2-Axis,Z_2-Axis', comments='')
