# Supplementary material for: The hunter and the hunted—A 3D analysis of predator-prey interactions between three-spined sticklebacks (Gasterosteus aculeatus) and larvae of different prey fishes
Source: PLoS One. 2021 Aug 26;16(8):e0256427. doi: 10.1371/journal.pone.0256427 (PMC8389440; doi:10.1371/journal.pone.0256427)
Supplement: S2 Appendix — (DOCX) [file pone.0256427.s002.docx]

**S2 Appendix. Python code (opened in jupyter notebook (5.1.0rc1)) used for calculating performance data from three-dimensional coordinates of tracked animals.**

#the relevant python packages are imported

import pandas as pd

import numpy as np

from os import listdir

path='file_path'

files_all=listdir(path)

(files_all)

files=list(filter(lambda x:'.csv' in x, files_all))

files

i=x #select file number x

track=pd.read_csv(path+files[i])

track['species']=files[i][0]

track['lenght']=files[i][2]

track['replicate']=files[i][4]

track['success']=files[i][6]

vx=track['X_1-Axis'].diff()

vy=track['Y_1-Axis'].diff()

vz=track['Z_1-Axis'].diff()

track['distance']=np.multiply(100,np.sqrt(np.square(vx)+np.square(vy)+np.square(vz)))

track['speed']=140*track['distance']

track['vx']=vx

track['turningangle']=np.degrees(np.arccos(np.divide(vx*vx.shift(-1)+vy*vy.shift(-1)+vz*vz.shift(-1),(np.sqrt(np.square(vx)+np.square(vy)+np.square(vz)))*(np.sqrt(np.square(vx.shift(-1))+np.square(vy.shift(-1))+np.square(vz.shift(-1)))))))

track['angular_velocity']=track['turningangle']*140/1000

track['distance_p_p']=np.multiply(100,np.sqrt(np.square(track['X_1-Axis']-track['X_2-Axis'])+np.square(track['Y_1-Axis']-track['Y_2-Axis'])+np.square(track['Z_1-Axis']-track['Z_2-Axis'])))

track['speed_pred']=np.multiply(14000,np.sqrt(np.square(track['X_2-Axis'].diff())+np.square(track['Y_2-Axis'].diff())+np.square(track['Z_2-Axis'].diff())))
