# Supplementary material for: The hunter and the hunted—A 3D analysis of predator-prey interactions between three-spined sticklebacks (Gasterosteus aculeatus) and larvae of different prey fishes
Source: PLoS One. 2021 Aug 26;16(8):e0256427. doi: 10.1371/journal.pone.0256427 (PMC8389440; doi:10.1371/journal.pone.0256427)
Supplement: S2 Fig — The black triangles mark the start of both tracks, while the black circles mark the end. (DOCX) [file pone.0256427.s004.docx]

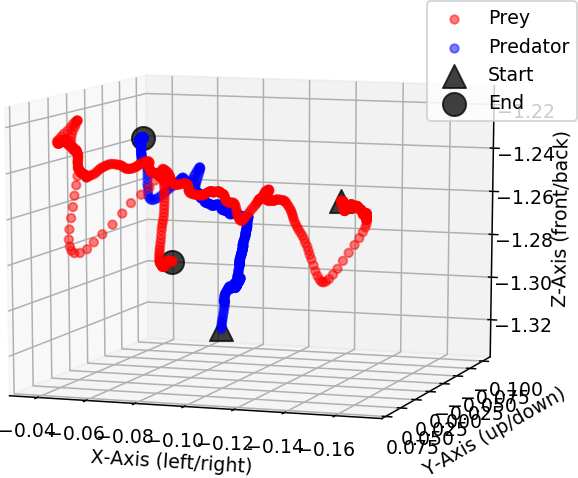


S2 Fig: 3D-Tracks of a failed hunt between a roach larva (red) and a stickleback (blue). The black triangles mark the start of both tracks, while the black circles mark the end.
