# Supplementary material for: The hunter and the hunted—A 3D analysis of predator-prey interactions between three-spined sticklebacks (Gasterosteus aculeatus) and larvae of different prey fishes
Source: PLoS One. 2021 Aug 26;16(8):e0256427. doi: 10.1371/journal.pone.0256427 (PMC8389440; doi:10.1371/journal.pone.0256427)
Supplement: S1 Table — (DOCX) [file pone.0256427.s006.docx]

**S1 Table. Number of evaluated failed and successful predator-prey interactions per prey species and size class.**

| **Species** | **Size class** | **Successful interaction** | **Failed interaction** |  |
| --- | --- | --- | --- | --- |
| Perch | 1 | 1 | 4 |  |
|  | 2 | 0 | 5 |  |
|  | 3 | 0 | 6 | larvae parameter only |
|  | 4 | 0 | 6 | larvae parameter only |
| Roach | 1 | 2 | 5 |  |
|  | 2 | 2 | 6 |  |
|  | 3 | 0 | 6 |  |
|  | 4 | 0 | 6 | larvae parameter only |
| Whitefish | 1 | 6 | 6 |  |
|  | 2 | 6 | 6 |  |
|  | 3 | 6 | 6 |  |
|  | 4 | 2 | 6 |  |
| Total: 75 predator-prey interactions. Additionally 18 failed interactions of escaping perch and roach larvae were included. | | | | |
