# Supplementary material for: The hunter and the hunted—A 3D analysis of predator-prey interactions between three-spined sticklebacks (Gasterosteus aculeatus) and larvae of different prey fishes
Source: PLoS One. 2021 Aug 26;16(8):e0256427. doi: 10.1371/journal.pone.0256427 (PMC8389440; doi:10.1371/journal.pone.0256427)
Supplement: S2 Table — The (†) below the species marks, what N was used for the corresponding performance variable per column. (DOCX) [file pone.0256427.s007.docx]

**S2 Table. Performance characteristics of predator and prey stratified by hunting outcome and prey species. The (†) below the species marks, what N was used for the corresponding performance variable per column.**

| **hunting outcome:** | failed hunt | | | successful hunt | | |
| --- | --- | --- | --- | --- | --- | --- |
| **prey species:** | Perch | Whitefish | Roach | Perch | Whitefish | Roach |
| performance variable: | N = 9; †N = 21 | N = 24 | N = 17; †N = 23 | N = 1 | N = 20 | N = 4 |
| Start hunt (s) (time before bite) | 1.9 ± 0.7 | 1.4 ± 0.7 | 1.5 ± 0.8 | 1.6 ± 0.0 | 1.3 ± 0.7 | 1.0 ± 1.0 |
| Dist. P-P start (cm) | 7.5 ± 4.2 | 8.6 ± 4.6 | 7.4 ± 6.9 | 12.2 ± 0.0 | 6.9 ± 4.3 | 6.1 ± 7.4 |
| Min. Dist. P-P (cm) | 2.6 ± 1.2 | 1.9 ± 1.4 | 1.4 ± 0.8 | 3.7 ± 0.0 | 1.3 ± 0.8 | 1.5 ± 0.4 |
| Speed Prey (cm/s) | 10.7 ± 6.0† | 11.1 ± 5.9 | 13.4 ± 7.6† | 5.7 ± 0.0 | 6.3 ± 3.4 | 9.7 ± 4.9 |
| Speed Pred. (cm/s) | 6.9 ± 2.1 | 13.7 ± 4.9 | 11.8 ± 4.6 | 10.0 ± 0.0 | 10.3 ± 3.6 | 14.2 ± 7.1 |
| Max. Speed Prey (cm/s) | 39.3 ± 18.4† | 45.8 ± 19.1 | 56.9 ± 28.8† | 30.0 ± 0.0 | 21.8 ± 17.0 | 32.4 ± 23.0 |
| Max. Speed Pred. (cm/s) | 31.9 ± 15.0 | 36.2 ± 16.5 | 50.7 ± 21.2 | 29.2 ± 0.0 | 27.7 ± 12.8 | 40.2 ± 24.4 |
| Acc. Prey (cm/s²) | 0.085 ± 0.108 | 0.194 ± 0.245 | 0.239 ± 0.224 | 0.002 ± 0.000 | -0.050 ± 0.060 | -0.217 ± 0.348 |
| Acc. Pred. (cm/s²) | 0.031 ± 0.043 | 0.042 ± 0.117 | 0.204 ± 0.465 | 0.029 ± 0.000 | -0.041 ± 0.066 | -0.380 ± 0.631 |
| Max. Acc. Prey (cm/s²) | 11.2 ± 6.1 | 12.9 ± 5.3 | 16.7 ± 6.9 | 7.2 ± 0.0 | 6.9 ± 4.8 | 9.3 ± 6.9 |
| Max. Acc. Pred. (cm/s²) | 11.4 ± 3.9 | 13.5 ± 7.8 | 15.5 ± 9.3 | 4.3 ± 0.0 | 10.6 ± 7.5 | 12.1 ± 6.3 |
| Turning angle Prey (°) | 15.0 ± 5.1† | 14.3 ± 5.3 | 13.0 ± 5.5† | 15.0 ± 0.0 | 15.5 ± 3.8 | 12.5 ± 3.5 |
| Turning angle Pred. (°) | 19.9 ± 2.5 | 15.5 ± 4.5 | 15.5 ± 6.1 | 7.5 ± 0.0 | 17.5 ± 5.5 | 19.0 ± 7.9 |
| Max. Turning angle Prey (°) | 113.2 ± 49.6† | 109.7 ± 37.6 | 99.4 ± 37.9† | 171.8 ± 0.0 | 92.1 ± 42.6 | 85.1 ± 59.9 |
| Max. Turning angle Pred. (°) | 133.9 ± 16.5 | 88.8 ± 28.2 | 112.8 ± 32.7 | 83.7 ± 0.0 | 105.6 ± 39.3 | 94.1 ± 26.4 |
