# Supplementary material for: The hunter and the hunted—A 3D analysis of predator-prey interactions between three-spined sticklebacks (Gasterosteus aculeatus) and larvae of different prey fishes
Source: PLoS One. 2021 Aug 26;16(8):e0256427. doi: 10.1371/journal.pone.0256427 (PMC8389440; doi:10.1371/journal.pone.0256427)
Supplement: S3 Table — (DOCX) [file pone.0256427.s008.docx]

**S3 Table. Statistical outcome of univariate logistic models of performance variables (left to right): roach, comparison between failed and successful hunts, corrected for trial (predator subject) and larvae size class (random effect); whitefish, comparison between failed and successful hunts, corrected for trial and size class; comparison between species within failed predations, corrected for size class; overall failed and successful hunts, corrected for predator and size (species nested)**

| factor comparison: | hunting success on roach larvae | hunting success on whitefish larvae | species differences within failed hunts | overall hunting success |  |
| --- | --- | --- | --- | --- | --- |
| random effect: | [Trial;Size] | [Trial;Size] | [Size] | [Trial;Size;Species nested] |  |
| Start hunt (s) | F(1,14.2) = 0.46; p = 0.510 | F(1,21.8) = 0.26; p = 0.616 | F(2,45.0) = 1.17; p = 0.320 | F(3,43.1) = 0.42; p = 0.737 |  |
| Dist. P-P Start (cm) | F(1,11.8) = 0.31; p = 0.588 | F(1,22.1) = 1.40; p = 0.250 | F(2,42.4) = 0.43; p = 0.653 | F(3,54.5) = 1.27; p = 0.292 |  |
| Min. Dist. P-P (cm) | F(1,18.7) = 0.00; p = 0.952 | F(1,22.0) = 2.63; p = 0.119 | F(2,40.5) = 2.10; p = 0.136 | F(3,50.7) = 1.17; p = 0.331 |  |
| Speed Prey (cm/s) | F(1,14.1) = 0.24; p = 0.629 | **F(1,21.3) = 4.67; p = 0.042** | F(2,62.2) = 1.21; p = 0.305 | F(3,48.7) = 2.23; p = 0.096 |  |
| Speed Pred. (cm/s) | **F(1,20.8) = 5.43; p = 0.030** | **F(2,34.3) = 6.35; p = 0.004** | F(3,52.0) = 2.35; p = 0.083 |  |  |
| Max. Speed Prey (cm/s) | **F(1,12.3) = 5.08; p = 0.043** | **F(1,23.3) = 18.30; p < 0.001** | **F(2,62.4) = 3.38; p = 0.040** | **F(3,52.0) = 7.76; p < 0.001** |  |
| Max. Speed Pred. (cm/s) | F(1,12.1) = 0.34; p = 0.570 | F(1,22.2) = 3.12; p = 0.091 | **F(2,44.7) = 4.07; p = 0.024** | F(3,46.7) = 1.18; p = 0.327 |  |
| Acc. Prey (cm/s²) | F(1,9.5) = 2.46; p = 0.149 | **F(1,21.6) = 8.37; p = 0.009** | F(2,41.5) = 1.92; p = 0.159 | **F(3,12.4) = 4.73; p = 0.020** |  |
| Acc. Pred. (cm/s²) | **F(1,15.3) = 9.91; p = 0.007** | **F(1,21.6) = 21.73; p < 0.001** | F(2,46.5) = 1.37; p = 0.264 | **F(3,45.9) = 12.32; p < 0.001** |  |
| Max. Acc. Prey (cm/s²) | **F(1,19.2) = 14.23; p = 0.001** | **F(2,36.7) = 3.65; p = 0.036** |  |  |  |
| Max. Acc. Pred. (cm/s²) | F(1,3.3) = 3.18; p = 0.165 | F(1,19.0) = 1.14; p = 0.300 | F(2,41.4) = 0.63; p = 0.536 | F(3,35.3) = 0.95; p = 0.428 |  |
| Turning angle Prey (°) | F(1,6.0) = 0.00; p = 0.962 | F(1,20.0) = 1.11; p = 0.304 | F(2,62.3) = 0.79; p = 0.456 | F(3,41.7) = 0.25; p = 0.864 |  |
| Turning angle Pred. (°) | F(1,19.9) = 3.22; p = 0.088 | **F(2,45.2) = 7.02; p = 0.002** | **F(3,41.6) = 3.28; p = 0.030** |  |  |
| Max. Turning angle Prey (°) | F(1,24.6) = 0.22; p = 0.640 | F(1,23.8) = 1.15; p = 0.293 | F(2,62.0) = 0.89; p = 0.414 | F(3,70.4) = 1.66; p = 0.184 |  |
| Max. Turning angle Pred. (°) | F(1,3.8) = 1.52; p = 0.289 | F(1,23.1) = 1.81; p = 0.191 | **F(2,41.9) = 9.05; p = 0.001** | F(3,56.5) = 1.97; p = 0.130 |  |
