# Supplementary material for: The hunter and the hunted—A 3D analysis of predator-prey interactions between three-spined sticklebacks (Gasterosteus aculeatus) and larvae of different prey fishes
Source: PLoS One. 2021 Aug 26;16(8):e0256427. doi: 10.1371/journal.pone.0256427 (PMC8389440; doi:10.1371/journal.pone.0256427)
Supplement: S4 Table — The (†) below the size marks, what N was used for the corresponding performance variable per column. (DOCX) [file pone.0256427.s009.docx]

**S4 Table. Performance characteristics of sticklebacks and perch, stratified by size class of perch as prey in the failed predation trials.** **The (†) below the size marks, what N was used for the corresponding performance variable per column.**

| Species | Perch |  |  |  |
| --- | --- | --- | --- | --- |
| Size | 1 | 2 | 3 | 4 |
|  | N = 4 | N = 6 | †N = 6 | †N = 6 |
| Start hunt (s) | 2.1 ± 0.4 | 1.7 ± 0.8 | n.d. | n.d. |
| Dist. P-P Start (cm) | 8.3 ± 6.5 | 6.9 ± 1.5 | n.d. | n.d. |
| Min. Dist. P-P (cm) | 2.5 ± 1.3 | 2.7 ± 1.3 | n.d. | n.d. |
| Speed Prey (cm/s) | 5.3 ± 2.4 | 5.2 ± 2.5 | 15.8 ± 5.8† | 13.5 ± 2.4† |
| Speed Pred. (cm/s) | 5.8 ± 2.3 | 7.8 ± 1.8 | n.d. | n.d. |
| Max. Speed Prey (cm/s) | 36.2 ± 21.6 | 38.2 ± 25.3 | 47.3 ± 16.6† | 34.3 ± 13.0† |
| Max. Speed Pred. (cm/s) | 35.2 ± 20.4 | 29.3 ± 10.9 | n.d. | n.d. |
| Acc. Prey (cm/s²) | 0.06 ± 0.06 | 0.11 ± 0.14 | n.d. | n.d. |
| Acc. Pred. (cm/s²) | 0.04 ± 0.07 | 0.02 ± 0.02 | n.d. | n.d. |
| Max. Acc. Prey (cm/s²) | 10.3 ± 3.7 | 11.9 ± 8.0 | n.d. | n.d. |
| Max. Acc. Pred. (cm/s²) | 11.2 ± 4.2 | 11.5 ± 4.2 | n.d. | n.d. |
| Turning angle Prey (°) | 17.6 ± 5.1 | 19.9 ± 4.2 | 14.3 ± 2.6† | 9.8 ± 2.2† |
| Turning angle Pred. (°) | 19.0 ± 3.7 | 20.5 ± 1.0 | n.d. | n.d. |
| Max. Turning angle Prey (°) | 168.4 ± 20.4 | 151.2 ± 20.6 | 96.2 ± 40.0† | 61.5 ± 14.1† |
| Max. Turning angle Pred. (°) | 134.9 ± 11.5 | 133.0 ± 21.0 | n.d. | n.d. |
