# Supplementary material for: The hunter and the hunted—A 3D analysis of predator-prey interactions between three-spined sticklebacks (Gasterosteus aculeatus) and larvae of different prey fishes
Source: PLoS One. 2021 Aug 26;16(8):e0256427. doi: 10.1371/journal.pone.0256427 (PMC8389440; doi:10.1371/journal.pone.0256427)
Supplement: S5 Table — (DOCX) [file pone.0256427.s010.docx]

**S5 Table. Performance characteristics of sticklebacks and whitefish, stratified by size class of whitefish as prey in the failed predation trials.**

| Species | Whitefish |  |  |  |
| --- | --- | --- | --- | --- |
| Size | 1 | 2 | 3 | 4 |
|  | N = 6 | N = 6 | N = 6 | N = 6 |
| Start hunt (s) | 1.4 ± 1.0 | 1.6 ± 0.8 | 1.1 ± 0.4 | 1.5 ± 0.7 |
| Dist. P-P Start (cm) | 9.3 ± 7.8 | 6.2 ± 2.4 | 9.0 ± 1.3 | 10.0 ± 4.4 |
| Min. Dist. P-P (cm) | 1.7 ± 1.6 | 2.1 ± 0.8 | 2.4 ± 2.0 | 1.3 ± 1.0 |
| Speed Prey (cm/s) | 12.3 ± 5.9 | 13.3 ± 5.3 | 12.0 ± 7.3 | 6.8 ± 3.9 |
| Speed Pred. (cm/s) | 14.2 ± 4.8 | 14.1 ± 5.6 | 14.4 ± 5.2 | 12.1 ± 5.2 |
| Max. Speed Prey (cm/s) | 45.7 ± 12.0 | 54.4 ± 21.8 | 47.2 ± 24.6 | 35.9 ± 15.6 |
| Max. Speed Pred. (cm/s) | 34.7 ± 14.9 | 40.4 ± 25.3 | 31.6 ± 13.4 | 38.1 ± 12.7 |
| Acc. Prey (cm/s²) | 0.31 ± 0.36 | 0.17 ± 0.22 | 0.13 ± 0.19 | 0.17 ± 0.19 |
| Acc. Pred. (cm/s²) | 0.03 ± 0.12 | 0.04 ± 0.04 | 0.00 ± 0.19 | 0.09 ± 0.07 |
| Max. Acc. Prey (cm/s²) | 13.4 ± 3.0 | 12.1 ± 5.7 | 13.6 ± 5.8 | 12.6 ± 7.2 |
| Max. Acc. Pred. (cm/s²) | 10.7 ± 5.2 | 14.6 ± 9.8 | 10.3 ± 4.6 | 18.4 ± 9.1 |
| Turning angle Prey (°) | 14.5 ± 4.8 | 10.4 ± 3.6 | 15.0 ± 5.9 | 17.4 ± 5.2 |
| Turning angle Pred. (°) | 12.3 ± 1.5 | 13.5 ± 3.3 | 15.2 ± 3.8 | 20.9 ± 3.7 |
| Max. Turning angle Prey (°) | 131.7 ± 31.4 | 87.2 ± 26.5 | 103.0 ± 46.4 | 116.7 ± 37.1 |
| Max. Turning angle Pred. (°) | 85.3 ± 41.1 | 89.5 ± 17.0 | 82.3 ± 35.3 | 98.2 ± 15.9 |
