# Supplementary material for: The hunter and the hunted—A 3D analysis of predator-prey interactions between three-spined sticklebacks (Gasterosteus aculeatus) and larvae of different prey fishes
Source: PLoS One. 2021 Aug 26;16(8):e0256427. doi: 10.1371/journal.pone.0256427 (PMC8389440; doi:10.1371/journal.pone.0256427)
Supplement: S6 Table — The (†) below the size marks, what N was used for the corresponding performance variable per column. (DOCX) [file pone.0256427.s011.docx]

**S6 Table.** **Performance characteristics of sticklebacks and roach, stratified by size class of roach as prey in the failed predation trials. The (†) below the size marks, what N was used for the corresponding performance variable per column.**

| Species | Roach |  |  |  |
| --- | --- | --- | --- | --- |
| Size | 1 | 2 | 3 | 4 |
|  | N = 6 | N = 6 | N = 6 | †N = 6 |
| Start hunt (s) | 1.4 ± 0.7 | 1.9 ± 0.7 | 1.3 ± 1.0 | n.d. |
| Dist. P-P Start (cm) | 6.0 ± 2.1 | 7.0 ± 4.9 | 9.0 ± 10.9 | n.d. |
| Min. Dist. P-P (cm) | 1.3 ± 0.4 | 1.1 ± 0.6 | 1.8 ± 1.2 | n.d. |
| Speed Prey (cm/s) | 11.8 ± 5.2 | 10.8 ± 7.6 | 9.4 ± 3.7 | 21.4 ± 7.2† |
| Speed Pred. (cm/s) | 14.2 ± 3.8 | 11.1 ± 4.9 | 10.6 ± 5.1 | n.d. |
| Max. Speed Prey (cm/s) | 74.9 ± 47.6 | 54.0 ± 28.5 | 50.8 ± 8.2 | 50.8 ± 23.1† |
| Max. Speed Pred. (cm/s) | 61.3 ± 22.1 | 51.3 ± 19.4 | 41.2 ± 21.2 | n.d. |
| Acc. Prey (cm/s²) | 0.37 ± 0.24 | 0.09 ± 0.14 | 0.28 ± 0.22 | n.d. |
| Acc. Pred. (cm/s²) | 0.18 ± 0.07 | 0.05 ± 0.06 | 0.38 ± 0.79 | n.d. |
| Max. Acc. Prey (cm/s²) | 15.3 ± 6.8 | 17.6 ± 7.9 | 17.0 ± 6.8 | n.d. |
| Max. Acc. Pred. (cm/s²) | 16.0 ± 8.2 | 16.3 ± 6.5 | 14.3 ± 13.2 | n.d. |
| Turning angle Prey (°) | 13.6 ± 4.0 | 10.2 ± 5.6 | 18.0 ± 6.1 | 10.4 ± 1.7† |
| Turning angle Pred. (°) | 14.0 ± 1.2 | 14.1 ± 7.0 | 18.2 ± 7.5 | n.d. |
| Max. Turning angle Prey (°) | 120.6 ± 34.1 | 98.5 ± 51.1 | 108.3 ± 36.0 | 73.7 ± 12.1† |
| Max. Turning angle Pred. (°) | 103.0 ± 33.8 | 120.5 ± 22.3 | 113.2 ± 42.9 | n.d. |
