# Supplementary material for: The hunter and the hunted—A 3D analysis of predator-prey interactions between three-spined sticklebacks (Gasterosteus aculeatus) and larvae of different prey fishes
Source: PLoS One. 2021 Aug 26;16(8):e0256427. doi: 10.1371/journal.pone.0256427 (PMC8389440; doi:10.1371/journal.pone.0256427)
Supplement: S7 Table — Only failed hunts were considered due to low n-values of successful hunts of perch and roach. (DOCX) [file pone.0256427.s012.docx]

**S7 Table. Outcome of the statistical comparison of the effect of size class on performance characteristics stratified for the different prey fish species. Only failed hunts were considered due to low n-values of successful hunts of perch and roach.**

|  | Perch | Whitefish | Roach |
| --- | --- | --- | --- |
| Start hunt (s) | F(1,0.4) = 0.87; p = 0.382 | F(3,1.0) = 0.59; p = 0.627 | F(2,1.4) = 1.02; p = 0.387 |
| Dist. P-P Start (cm) | F(1,4.5) = 0.23; p = 0.644 | F(3,48.7) = 0.74; p = 0.538 | F(2,25.6) = 0.25; p = 0.784 |
| Min. Dist. P-P (cm) | F(1,0.1) = 0.04; p = 0.851 | F(3,3.6) = 0.58; p = 0.632 | F(2,1.6) = 1.27; p = 0.312 |
| Speed Prey (cm/s) | **F(3,471.2) = 11.00; p < 0.001** | F(3,153.3) = 1.56; p = 0.230 | **F(3,530.9) = 4.64; p = 0.014** |
| Speed Pred. (cm/s) | F(1,9.0) = 2.26; p = 0.177 | F(3,19.7) = 0.24; p = 0.865 | F(2,39.0) = 0.89; p = 0.431 |
| Max. Speed Prey (cm/s) | F(3,580.7) = 0.53; p = 0.667 | F(3,1040.6) = 0.95; p = 0.437 | F(3,2113.1) = 0.83; p = 0.494 |
| Max. Speed Pred. (cm/s) | F(1,77.0) = 0.31; p = 0.594 | F(3,266.0) = 0.30; p = 0.828 | F(2,1103.2) = 1.27; p = 0.311 |
| Acc. Prey (cm/s²) | F(1,0.0) = 0.42; p = 0.536 | F(3,0.0) = 0.63; p = 0.607 | F(2,0.3) = 0.73; p = 0.499 |
| Acc. Pred. (cm/s²) | F(1,0.0) = 0.36; p = 0.567 | F(3,0.1) = 0.61; p = 0.614 | F(2,0.2) = 2.90; p = 0.088 |
| Max. Acc. Prey (cm/s²) | F(1,5.4) = 0.13; p = 0.731 | F(3,9.1) = 0.10; p = 0.962 | F(2,14.8) = 0.14; p = 0.870 |
| Max. Acc. Pred. (cm/s²) | F(1,0.2) = 0.01; p = 0.920 | F(3,259.9) = 1.52; p = 0.239 | F(2,13.9) = 0.07; p = 0.931 |
| Turning angle Prey (°) | **F(3,314.8) = 8.56; p = 0.001** | F(3,155.7) = 2.14; p = 0.127 | **F(3,236.3) = 3.56; p = 0.034** |
| Turning angle Pred. (°) | F(1,5.1) = 0.79; p = 0.404 | **F(3,266.2) = 8.55; p = 0.001** | F(2,66.9) = 0.88; p = 0.435 |
| Max. Turning angle Prey (°) | **F(3,37170.6) = 17.63; p < 0.001** | F(3,6505.5) = 1.66; p = 0.207 | F(3,6665.8) = 1.69; p = 0.202 |
| Max. Turning angle Pred. (°) | F(1,8.0) = 0.03; p = 0.877 | F(3,865.2) = 0.33; p = 0.802 | F(2,835.6) = 0.36; p = 0.704 |
